# Supplementary material for: Multiple Human Papilloma Virus (HPV) Infections Are Associated with HSIL and Persistent HPV Infection Status in Korean Patients
Source: Viruses. 2021 Jul 12;13(7):1342. doi: 10.3390/v13071342 (PMC8310096; doi:10.3390/v13071342)
Supplement: Supplementary file 1 [file viruses-13-01342-s001.zip › suppplementary figures.pdf]

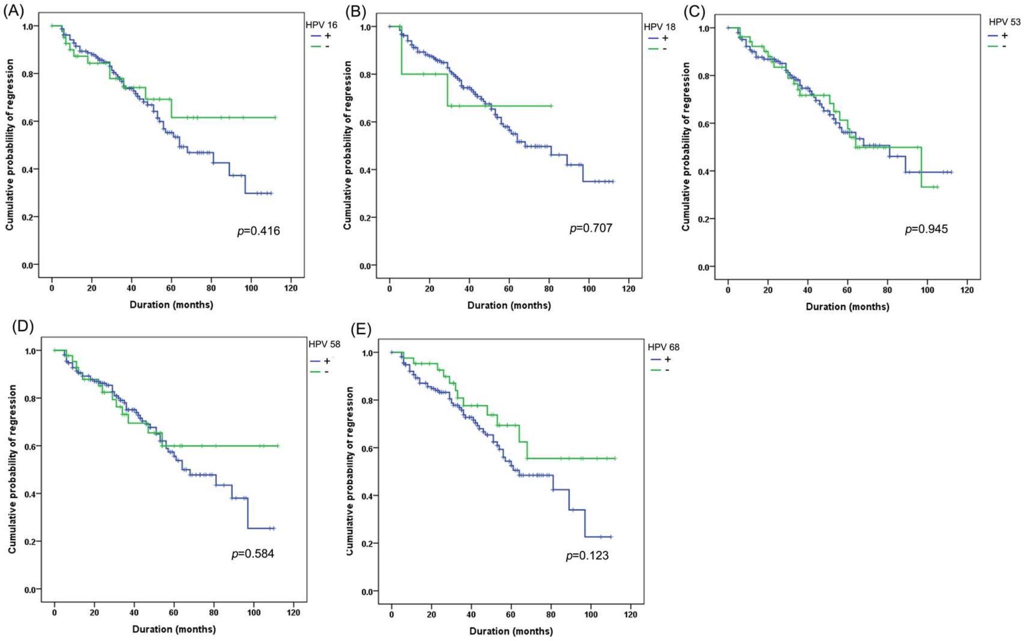

Figure S1: Regression or persistence of HPV infection during follow-up period according to HPV genotypes (in multiple HPV infection): (**A**) HPV 16; (**B**) HPV 18; (**C**) HPV 53; (**D**) HPV 58; and (**E**) HPV 68.

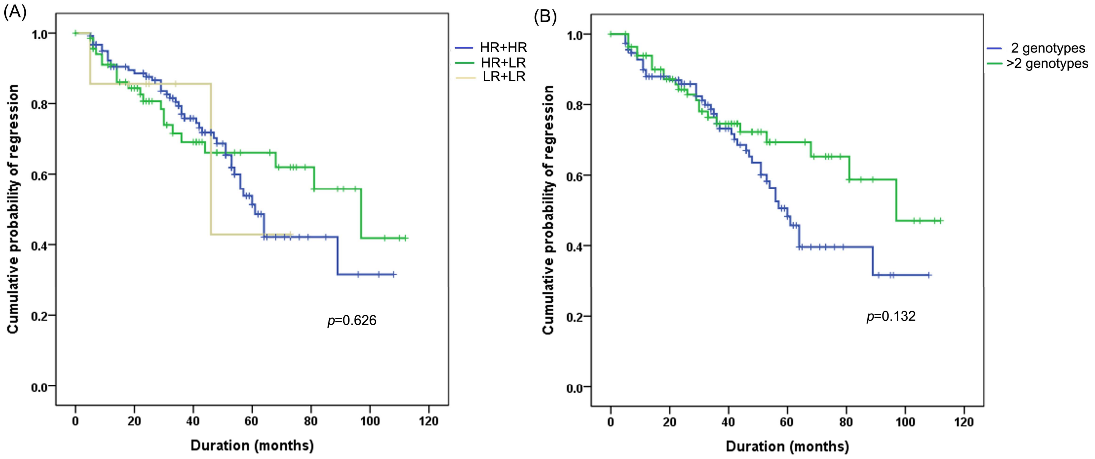

Figure S2: Regression or persistence of HPV infection during follow-up period according to HPV genotypes (in multiple HPV infection): (**A**) Combination patterns of HR- or LR-HPV and (**B**) number of HPV genotypes.
